# Analysis of Mechanical Parameters in Multi-Pass Asymmetrical Rolling of Strip by Slab Method

**DOI:** 10.3390/ma16186286

**Published:** 2023-09-19

**Authors:** Qilin Zhao, Xianlei Hu, Xianghua Liu

**Affiliations:** 1 School of Materials Science and Engineering, Northeastern University, Shenyang 110819, China; zhaoql73@163.com; 2 State Key Laboratory of Rolling and Automation, Northeastern University, Shenyang 110819, China

**Keywords:** multi-pass asymmetrical rolling, roll force, roll torque, roll power, rolling time, energy consumption, critical speed ratio, mathematic model, slab method

## Abstract

Mechanical parameters, time consumption and energy consumption are important considerations in the application of a certain rolling process. This study aims to investigate characteristics of the roll force, roll torque, roll power, rolling time and total work in multi-pass asymmetrical rolling of strip. Mathematic models were built using the slab method to calculate parameters in the asymmetrical rolling process, and the characteristics of these parameters were analyzed on the basis of simulation results. Mechanical parameters are affected by the change of deformation region type. When the speed ratio is less than the critical speed ratio, the roll force, absolute values of roll torque and roll power are found to increase with the increase in the speed ratio. After the speed ratio reaches the critical speed ratio, the roll force, roll torque and lower roll power keep constant, but the upper roll power continues increasing. The upper roll torque and upper roll power required by asymmetrical rolling are much greater than that by symmetrical rolling, which indicates that stronger drive shafts and more powerful drive motors are required by asymmetrical rolling. Compared with symmetrical rolling, asymmetrical rolling requires less roll force to obtain the same thickness reduction, especially for thin and hard strips. Rolling time can be saved at the cost of more energy consumption by using asymmetrical rolling with the same roll force to attain the same final thickness. The results and conclusions of this study can provide a reference for mill design and application of asymmetrical rolling in strip manufacturing.

## 1. Introduction

Asymmetrical rolling is a rolling process in which differential speed between the upper and the lower surface of the rolled piece is produced by different roll diameters, different roll speed or different friction conditions. For the advantages of low cost, simplicity and less force requirement, it has been extensively investigated since it was first introduced.

Since less roll force is required by asymmetrical rolling, numerous investigations were conducted on behaviors of a work piece in the deformation region and process parameters. Slab method [1] is the most commonly used analytical method. Huang et al. [2,3,4] studied the sheet behaviors and roll pressure distributions in asymmetrical rolling with the slab method, finite element method and stream function method. Salimi et al. [5] proposed a model considering the non-uniformity of the normal and shear stress profiles across the section of the product to calculate rolling force, torque and pressure in asymmetrical rolling. Tian et al. [6] built a model assuming that the contact arc was parabolic and the surface friction was constant to analyze the influence of asymmetrical rolling factors on the deformation area and unit pressure. Analytical models considering the vertical shear stress were developed by Zhang et al. [7] to calculate the roll force and torque. Razani et al. [8] established a new model considering the rate-dependent condition of yield shear stress using the slab method to calculate the rolling force in asymmetrical thermomechanical rolling.

Bending of the outgoing work piece is a common phenomenon in asymmetrical rolling, which always attracts the attention of researchers. A model based on the slab method was developed by Qwamizadeh et al. [9], which was used to calculate the strip curvature in asymmetrical sheet rolling. Salimi et al. [10] built a model considering the variation of the shear stress within the roll gap to predict the strip curvature. Aboutorabi et al. [11] proposed a formula considering the rolls horizontal displacement to predict the sheet curvature in the asymmetrical rolling. Mousavi et al. [12] investigated the effects of angular velocities on sheet curvature using the finite element method. Su et al. [13] studied the bending behavior and mechanism during the asymmetrical rolling processing of high-strength AA7050 aluminum alloy plates via simulation and rolling trials.

The outstanding capability of asymmetrical rolling in thickness reduction arouses interest in minimum thickness in asymmetrical rolling. Tzou et al. [14,15] also studied the minimum reliable thickness theoretically and experimentally in the hot-and-cold rolling and PV rolling considering different friction conditions. Tang et al. [16] investigated the minimum reliable thickness by introducing the percentage of the cross-shear zone. Feng et al. [17] analyzed the minimum thickness of single-roll-driven asymmetric rolling theoretically with finite element models and theoretical analytical models.

Since asymmetrical rolling can provide different speeds on the upper and lower surfaces of the work piece, it is also employed in the research work of composite plates and multilayer materials. Assuming the ingoing sheet is guided to horizontally enter the roll gap, Qwamizadeh and his coworkers [18,19] analyzed the asymmetrical rolling of bonded two-layer clad sheets with the slab method. Afrouz and Parvizi [20] built a model with the slab method to investigate the effects of the process parameters on the process outputs of asymmetric rolling of unbonded clad sheets. Wang et al. [21] proposed a model considering the vertical stresses to calculate the rolling force and torque in asymmetrical rolling of unbonded two-layer clad sheets. In the study on microstructure, interface structure and mechanical properties of composite plates, Zhi et al. [22] employed asymmetrical rolling to prepare Mg/Al composite plates with different thickness ratios.

Due to its capability to form a large collection of texture components, which is beneficial to the mechanical properties of metallic materials, asymmetrical rolling is increasingly employed in the research works of microstructure evolution, grain refinement and enhancement of the mechanical properties of materials. Nam et al. [23] increased the R-value of an AA1050 Al alloy sheet from 0.61 to 1.3 using asymmetrical rolling and heat treatment. Dhinwal et al. [24] studied the influence of thickness reduction on texture evolutions in asymmetric rolling of extra-low-carbon steel. Combination Models were built by Zhang et al. [25] to study the dynamic recrystallization of plates during asymmetrical rolling. The asymmetrical rolling experiment was conducted by Amegadzie et al. [26] to investigate influence factors on the mechanical properties of aluminum alloy AA6061, especially the effects of processing parameters. Lin et al. [27] investigated the effects of different speed ratios on the homogeneity of through thickness microstructure and texture. Li et al. [28] explored the microstructure and mechanical properties of a medium-carbon low-alloy steel prepared via the asymmetric rolling process with different speed ratios and suggested that strengthening is mainly because of the introduction of extra shear stress on the edge under asymmetric rolling. In the exploration of the mechanical properties of pure titanium foils, asymmetrical rolling was employed by Xiao et al. [29] in the grain refinement process to produce compressive strain and shear strain.

Though extensive research has been conducted on various aspects of asymmetrical rolling, studies on characteristics of multi-pass rolling processes are seldom reported. Characteristics of mechanical parameters (such as roll force, roll torque and roll power) in different deformation region types, different passes, and time and energy consumptions in multi-pass rolling are very important to the application of asymmetrical rolling in strip manufacturing. In this paper, analytical models are built with the slab method to calculate three zone percentages, the critical speed ratio, roll force, roll torque and power in multi-pass asymmetrical rolling of the strip. From the calculated results, characteristics of roll force, roll torque and roll power are analyzed, as well as the total rolling time and work consumption required to attain a certain final thickness. Experimental rolling is conducted to verify the accuracy of proposed models, and good agreement is found between the experiment results and the analysis values.

## 2. Mathematical Models

**Figure 1:**
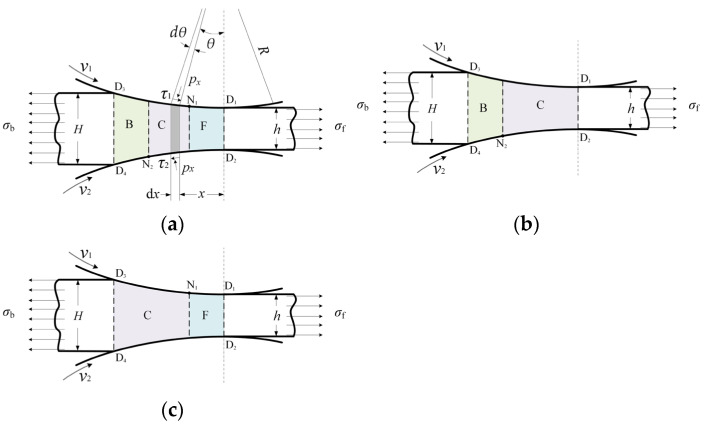
Schematic of deformation region types in asymmetrical rolling of strip: (**a**) Type I, (**b**) Type II, (**c**) Type III.

Figure 1a shows a typical deformation region in the asymmetrical rolling of strip. As can be seen from the figure, the deformation region is divided by the upper neutral point (N_1_) and the lower neutral point (N_2_) into forward-slip zone (F), cross-shear zone (C) and backward-slip zone (B). The change of relative speed between roll surface and work piece results in movements of neutral points along the contact arc and, consequently, changing percentages of the three zones. When N_1_ moves to the exit of the deformation region, the forward-slip zone disappears. When N_2_ moves to the entry of the deformation region, the backward-slip zone disappears. Three types of deformation region are defined according to zones consisting the deformation region. Type I consists of all three zones (Figure 1a), Type II consists of cross-shear zone and backward-slip zone (Figure 1b), and Type III consists of forward-slip zone and cross-shear zone (Figure 1c). For the convenience of description, Zone B, Zone C and Zone F are used to denote the backward-slip zone, the cross-shear zone and the forward-slip zone, respectively.

### 2.1. Basic Assumptions

In the derivation of models for asymmetrical rolling of strip, the following assumptions are employed for simplicity:

(1) Rolls are rigid bodies with the same diameters, and the strips are made of rigid-plastic material.
(2) Coulomb friction with constant friction coefficient is assumed between strip and roll, but the friction coefficients on the upper and the lower surface of strip may be different.
(3) The plastic deformation of the strip is plane strain.
(4) The vertical stress and the horizontal stress are uniformly distributed within slabs, and they are regarded as principal stresses.
(5) The contact arc is assumed as string, and the projected contact lengths of upper roll and lower roll are equal.

### 2.2. Roll Pressure

Figure 1a shows the schematic of a typical deformation region in asymmetrical rolling of strip, which consists of Zone B, Zone C and Zone F. In the figure, *R* is the roll radius; $v_{1}$ and $v_{2}$ represent upper roll speed and lower roll speed, respectively, and $v_{1}\geq v_{2}$; *θ* is the angle from the plane of roll axes; *H* and *h* are entry thickness and exit thickness, respectively; *x* represents the distance from the exit plane D_1_D_2_.

**Figure 2:**
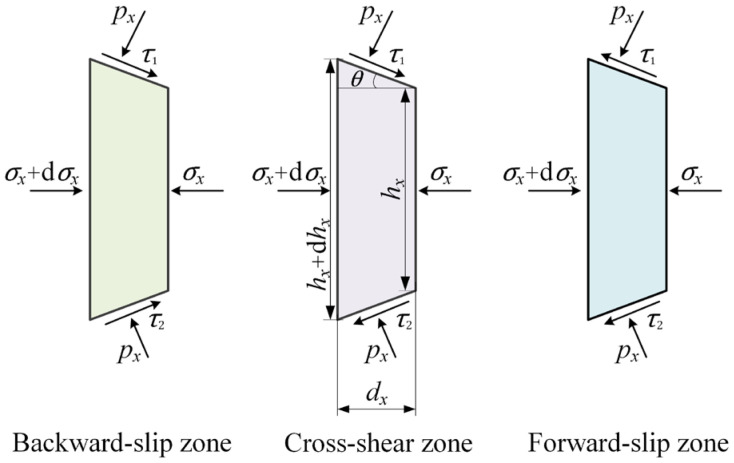
Stresses on slabs taken from different deformation zones.

Figure 2 shows stresses on slabs taken from three deformation zones of Figure 1a, where $p_{x}$ is the normal pressure; $\sigma_{x}$ is the horizontal stress; $\tau_{1}=\mu_{1}p_{x}$ and $\tau_{2}=\mu_{2}p_{x}$ are friction stresses between the strip and the upper and the lower rolls, $\mu_{1}$ and $\mu_{2}$ are friction coefficients; $h_{x}$ is the thickness of the strip at co-ordinate *x*.

From stresses on a slab (Figure 2), the equilibrium of horizontal forces on the slab can be obtained:

(1) $$(\sigma_{x}+d\sigma_{x})(h_{x}+dh_{x})-\sigma_{x}h_{x}-2p_{x}Rd\theta \sin\theta +\tau Rd\theta \cos\theta =0$$

where *τ* is the resultant friction stress, which is $\tau_{1}+\tau_{2}$, $\tau_{1}-\tau_{2}$ and $-\left(\tau_{1}+\tau_{2}\right)$ in Zone B, Zone C and Zone F, respectively.

In plane strain, the yield criterion is as follows:

(2) $$p_{x}-\sigma_{x}=K$$

where *K* is the plane deformation resistance of strip.

Referring to the geometry shown in Figure 1a, strip thickness at *x* is as follows:

(3) $$h_{x}=h+\frac{\Delta h}{l}x$$

where *l* is the contact length and Δh=H−h is the thickness reduction.

Substituting Equations (2) and (3) into Equation (1) gives the following:

(4) $$dp_{{}_{x}}-\frac{dh_{x}}{h_{x}}M=0$$

where $M=K-\delta^{+}p_{x}$ in Zone B, $M=K-\delta^{-}p_{x}$ in Zone C, $M=K+\delta^{+}p_{x}$ in Zone F; $\delta^{+}=\frac{(\mu_{1}+\mu_{2})l}{\Delta h}$ and $\delta^{-}=\frac{(\mu_{1}-\mu_{2})l}{\Delta h}$.

The solution of Equation (4) is obtained by integration:

(5) $$N-\ln\frac{1}{h_{x}}=C*$$

where C* is integral constant, which is different for Zone B, Zone C and Zone F; $N=\frac{1}{\delta^{+}}\ln\left(\delta^{+}p_{x}-K\right)$ in Zone B, $N=\frac{1}{\delta^{-}}\ln\left(\delta^{-}p_{x}-K\right)$ in Zone C and $N=\frac{1}{\delta^{+}}\ln\left(\delta^{+}p_{x}+K\right)$ in Zone F.

When the deformation region consists of only Zone B and Zone C (Type II), boundary conditions are $h_{x}=H$ and $p_{x}=K-\sigma_{b}$ on the entry plane, while $h_{x}=h$ and $p_{x}=K-\sigma_{f}$ on the exit plane. $\sigma_{b}$ and $\sigma_{f}$ are back tension and front tension, respectively. With boundary conditions, integral constants of Zone B and Zone C in Equation (5) can be determined; thus, the roll pressure in Zone B and Zone C is obtained:

(6) $$p_{B(\mathrm{II})}=\frac{K}{\delta^{+}}\left[(\delta^{+}\varepsilon_{b}-1){\left(\frac{H}{h_{x}}\right)}^{\delta^{+}}+1\right]$$

(7) $$p_{C(\mathrm{II})}=\frac{K}{\delta^{-}}\left[\left(\delta^{-}\varepsilon_{f}-1\right){\left(\frac{h}{h_{x}}\right)}^{\delta^{-}}+1\right]$$

where $\varepsilon_{b}=1-\sigma_{b}/K$ and $\varepsilon_{f}=1-\sigma_{f}/K$.

When there are only Zone C and Zone F in deformation region (Type III), the roll pressure of Zone C and Zone F is as follows:

(8) $$p_{C(\mathrm{III})}=\frac{K}{\delta^{-}}\left[(\delta^{-}\varepsilon_{b}-1){\left(\frac{H}{h_{x}}\right)}^{\delta^{-}}+1\right]$$

(9) $$p_{F(\mathrm{III})}=\frac{K}{\delta^{+}}\left[(\delta^{+}\varepsilon_{f}+1){\left(\frac{h_{x}}{h}\right)}^{\delta^{+}}-1\right]$$

When the deformation region consists of all three zones (Type I), roll pressures in Zone B and Zone F can be obtained from boundary conditions on the entry plane and exit plane:

(10) $$p_{B(I)}=\frac{K}{\delta^{+}}\left[(\delta^{+}\varepsilon_{b}-1){\left(\frac{H}{h_{x}}\right)}^{\delta^{+}}+1\right]$$

(11) $$p_{F(I)}=\frac{K}{\delta^{+}}\left[(\delta^{+}\varepsilon_{f}+1){\left(\frac{h_{x}}{h}\right)}^{\delta^{+}}-1\right]$$

On the interface between Zone B and Zone C, roll pressure of Zone B and Zone C is equal. Strip thickness is $h_{x}=H-Q_{B}\Delta h$, where $Q_{B}$ is the percentage of the backward-slip zone. The integral constant of Zone C is determined from the boundary conditions on the interface; then, the roll pressure of Zone C is obtained:

(12) $$p_{C(I)}=\frac{K}{\delta^{-}}{\left(\frac{H}{\xi_{B}h_{x}}\right)}^{\delta^{-}}\left[\frac{\delta^{-}}{\delta^{+}}\left(\delta^{+}\varepsilon_{b}-1\right)\xi_{B}^{\delta^{+}}+\frac{\delta^{-}}{\delta^{+}}-1\right]+\frac{K}{\delta^{-}}$$

where $\xi_{B}=H/\left(H-Q_{B}\Delta h\right)$.

### 2.3. Three Zone Percentages

**Figure 3:**
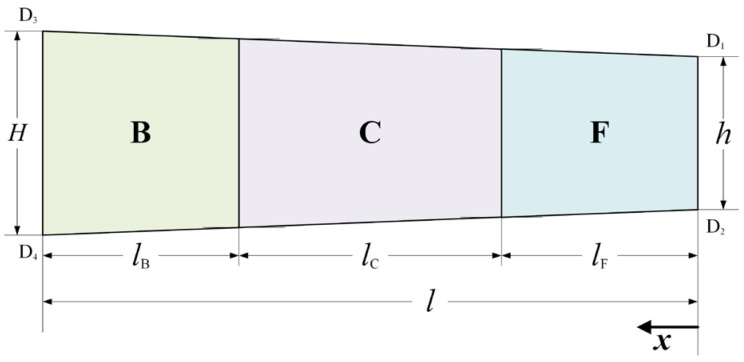
Schematic of the simplified geometry of the plastic deformation region in asymmetrical rolling of strip.

Figure 3 shows the schematic of the simplified geometry of the plastic deformation region in asymmetrical rolling of strip. $l_{B}$, $l_{C}$ and $l_{F}$ are projected lengths of Zone B, Zone C and Zone F, respectively, and percentages of these zones are defined as $Q_{B}=l_{B}/l$, $Q_{C}=l_{C}/l$ and $Q_{F}=l_{F}/l$.

Since roll pressure is successively distributed on the contact arc, the roll pressures of neighboring zones are equal at the interface between them.

On the interface between Zone B and Zone C in Type II, $p_{B(\mathrm{II})}=p_{C(\mathrm{II})}$ and $h_{x}=H-Q_{B}\Delta h$. From Equations (6) and (7), $Q_{B}$ and $Q_{C}$ of Type II can be obtained:

(13) $$Q_{B(\mathrm{II})}=\frac{H\left(\xi_{B}-1\right)}{\Delta h\xi_{B}}$$

(14) $$Q_{C(\mathrm{II})}=\frac{H-{\Delta h\xi }_{B}}{{\Delta h\xi }_{B}}$$

where $\xi_{B}$ can be obtained by iterative method via Equation (15):

(15) $$\delta^{-}\left(\delta^{+}\varepsilon_{b}-1\right)\mu^{\delta^{+}}\xi_{B}^{\delta^{+}}+\delta^{-}=\delta^{+}\left(\delta^{-}\varepsilon_{f}-1\right)\xi_{B}^{\delta^{-}}\xi^{-\delta^{-}}+\delta^{+}$$

where ξ=H/h.

By the same method, $Q_{C}$ and $Q_{F}$ of Type III can be obtained from Equations (16)–(18):

(16) $$Q_{C(\mathrm{III})}=\frac{H-h\xi_{F}}{\Delta h}$$

(17) $$Q_{F(\mathrm{III})}=\frac{h\xi_{F}-h}{\Delta h}$$

(18) $$\delta^{-}\left(\delta^{+}\varepsilon_{f}+1\right)\xi_{F}^{\delta^{+}}-\delta^{-}=\delta^{+}\left(\delta^{-}\varepsilon_{b}-1\right)\xi^{\delta^{-}}\xi_{F}^{\delta^{-}}+\delta^{+}$$

where $\xi_{F}=\left(h+Q_{F}\Delta h\right)/h$.

Percentages of Zone B, Zone C and Zone F in Type I are expressed, respectively, as follows:

(19) $$Q_{B(I)}=(H-HX^{-\frac{1}{\delta^{+}}})/\Delta h$$

(20) $$Q_{C(I)}=H(i-1)X^{-\frac{1}{\delta^{+}}}/(i\Delta h)$$

(21) $$Q_{F(I)}=(HX^{-\frac{1}{\delta^{+}}}-ih)/(i\Delta h)$$

where

(22) $$X=\frac{\sqrt{{\left(i^{\delta^{-}}-\frac{\delta^{+}}{\delta^{-}}i^{\delta^{-}}+\frac{\delta^{+}}{\delta^{-}}+1\right)}^{2}+4\left(\delta^{+}\varepsilon_{b}-1\right)\left(\delta^{+}\varepsilon_{f}+1\right)i^{\delta^{-}}{\left(\frac{\xi }{i}\right)}^{\delta^{+}}}-i^{\delta^{-}}+\frac{\delta^{+}}{\delta^{-}}i^{\delta^{-}}-\frac{\delta^{+}}{\delta^{-}}-1}{2\left(\delta^{+}\varepsilon_{b}-1\right)i^{\delta^{-}}}$$

### 2.4. Critical Speed Ratio

Three zone percentages of Type I vary with the speed ratio. When $Q_{F}$ becomes zero, $Q_{B}>0$ and $Q_{C}>0$, the deformation region changes from Type I to Type II. The speed ratio, at which Zone F disappears, is defined as the critical speed ratio of Type II, denoted by $i_{c(\mathrm{II})}$. On the other hand, when $Q_{B}$ becomes zero, $Q_{F}>0$ and $Q_{C}>0$, the critical speed ratio of Type III, $i_{c(\mathrm{III})}$, is reached.

When speed ratio is $i_{c(\mathrm{II})}$, $Q_{F(I)}=0$:

(23) $$Q_{F(I)}=\frac{HX^{-\frac{1}{\delta^{+}}}-i_{c(\mathrm{II})}h}{i_{c(\mathrm{II})}\Delta h}=0$$

Substituting Equation (22) into Equation (23), we have the following:

(24) $$\delta^{+}\varepsilon_{f}=i_{c(\mathrm{II})}^{\delta^{-}}\left(\delta^{+}\varepsilon_{b}-1\right){\left(\frac{\xi }{i_{c(\mathrm{II})}}\right)}^{\delta^{+}}+i_{c(\mathrm{II})}^{\delta^{-}}-\frac{\delta^{+}}{\delta^{-}}i_{c(\mathrm{II})}^{\delta^{-}}+\frac{\delta^{+}}{\delta^{-}}$$

From Equation (24), $i_{c(\mathrm{II})}$ can be obtained with the iterative method. Similarly, Equation (25) is obtained from Equation (19) and $i_{c(\mathrm{III})}$ can be obtained via iteration:

(25) $$\left(\delta^{+}\varepsilon_{f}+1\right){\left(\frac{\xi }{i_{c(\mathrm{III})}}\right)}^{\delta^{+}}=\delta^{+}\varepsilon_{b}i_{c(\mathrm{III})}^{\delta^{-}}-\frac{\delta^{+}}{\delta^{-}}i_{c(\mathrm{III})}^{\delta^{-}}+\frac{\delta^{+}}{\delta^{-}}+1$$

### 2.5. Roll Force

Integrating the normal roll pressure along the contact arc yields the roll force per unit width:

(26) $$P_{\left(I\right)}=\int_{0}^{l_{F}}p_{F(I)}dx+\int_{l_{F}}^{l_{F}+l_{C}}p_{C(I)}dx+\int_{l-l_{B}}^{l}p_{B(I)}dx$$

(27) $$P_{\left(\mathrm{II}\right)}=\int_{0}^{l_{C}}p_{C(\mathrm{II})}dx++\int_{l-l_{B}}^{l}p_{B(\mathrm{II})}dx$$

(28) $$P_{\left(\mathrm{III}\right)}=\int_{0}^{l_{F}}p_{F(\mathrm{III})}dx++\int_{l-l_{C}}^{l}p_{C(\mathrm{III})}dx$$

By substituting roll pressures into the above equations and integrating, the roll forces per unit width of three deformation region types are obtained, respectively, as follows:

(29) $$\begin{array}{cc} P_{\left(I\right)} & =\frac{Kl}{\delta^{+}}\left[\frac{H\left(\delta^{+}\varepsilon_{b}-1\right)\left(\xi_{B}^{\delta^{+}-1}-1\right)}{\Delta h\left(\delta^{+}-1\right)}+Q_{B}\right]+\frac{Kl}{\delta^{-}}\left\{\frac{\left(H-Q_{B}\Delta h\right)}{\Delta h\left(\delta^{-}-1\right)}\left({\left(\xi \xi_{B}\xi_{F}\right)}^{{1-\delta }^{-}}-1\right)\left[\frac{\delta^{-}}{\delta^{+}}\left(\delta^{+}\varepsilon_{b}-1\right)\xi_{B}^{\delta^{+}}+\frac{\delta^{-}}{\delta^{+}}-1\right]+Q_{C}\right\}\\ & +\frac{Kl}{\delta^{+}}\left[\frac{h\left(\delta^{+}\varepsilon_{f}+1\right)\left(\xi_{F}^{\delta^{+}+1}-1\right)}{\Delta h\left(\delta^{+}+1\right)}-Q_{F}\right] \end{array}$$

(30) $$P_{\left(\mathrm{II}\right)}=\frac{Kl}{\delta^{+}}\left[\frac{H\left(\delta^{+}\varepsilon_{b}-1\right)\left(\xi_{B}^{\delta^{+}-1}-1\right)}{\Delta h\left(\delta^{+}-1\right)}+Q_{B}\right]+\frac{Kl}{\delta^{-}}\left[\frac{h\left(\delta^{-}\varepsilon_{f}-1\right)\left({\left(\xi \xi_{B}\right)}^{\delta^{-}-1}-1\right)}{\Delta h\left(1-\delta^{-}\right)}+Q_{C}\right]$$

(31) $$P_{\left(\mathrm{III}\right)}=\frac{Kl}{\delta^{-}}\left[\frac{H\left(\delta^{-}\varepsilon_{b}-1\right)\left(\xi^{\delta^{-}-1}\xi_{F}^{{1-\delta }^{-}}-1\right)}{\Delta h\left(\delta^{-}-1\right)}+Q_{C}\right]+\frac{Kl}{\delta^{+}}\left[\frac{h\left(\delta^{+}\varepsilon_{f}+1\right)\left(\xi_{B}^{\delta^{+}+1}-1\right)}{\Delta h\left(\delta^{+}+1\right)}-Q_{F}\right]$$

### 2.6. Roll Torque

The total roll torque acting upon one is equal to the integration of the moment of the friction force about the roll axis along the contact arc. Thus, the upper roll torque per unit width $T_{1(I)}$ and the lower roll torque per unit width $T_{2(I)}$ of Type I can be obtained:

(32) $$T_{1(I)}=\int_{\gamma_{2}}^{\alpha }Rp_{B(I)}\mu_{1}Rd\theta +\int_{\gamma_{1}}^{\gamma_{2}}Rp_{C(I)}\mu_{1}Rd\theta -\int_{0}^{\gamma_{1}}Rp_{F(I)}\mu_{1}Rd\theta$$

(33) $$T_{2(I)}=\int_{\gamma_{2}}^{\alpha }Rp_{B(I)}\mu_{2}Rd\theta -\int_{\gamma_{1}}^{\gamma_{2}}Rp_{C(I)}\mu_{2}Rd\theta -\int_{0}^{\gamma_{1}}Rp_{F(I)}\mu_{2}Rd\theta$$

where $\gamma_{1}$ and $\gamma_{2}$ are neutral angles of the upper and lower contact arc respectively, α is the bite angle.

By substituting roll pressures for Type I, i.e., Equations (10)–(12), into Equations (32) and (33) and integrating, the roll torque can be obtained for upper and lower roll:

(34) $$T_{1(I)}=R\mu_{1}\left[P_{\left(I\right)}-\frac{2Klh\left(\delta^{+}\varepsilon_{f}+1\right)\left(\xi_{F}^{\delta^{+}+1}-1\right)}{\delta^{+}\Delta h\left(\delta^{+}+1\right)}+\frac{2KlQ_{F}}{\delta^{+}}\right]$$

(35) $$T_{2(I)}=R\mu_{2}\left[\frac{2KlH\left(\delta^{+}\varepsilon_{b}-1\right)\left(\xi_{B}^{\delta^{+}-1}-1\right)}{\delta^{+}\Delta h\left(\delta^{+}-1\right)}+\frac{2KlQ_{B}}{\delta^{+}}-P_{\left(I\right)}\right]$$

In the same way, roll torque for Type II can be expressed as follows:

(36) $$T_{1(\mathrm{II})}=R\mu_{1}P_{\left(\mathrm{II}\right)}$$

(37) $$T_{2(\mathrm{II})}=R\mu_{2}\left[\frac{2KlH\left(\delta^{+}\varepsilon_{b}-1\right)\left(\xi_{B}^{\delta^{+}-1}-1\right)}{\delta^{+}\Delta h\left(\delta^{+}-1\right)}+\frac{2KlQ_{B}}{\delta^{+}}-P_{\left(\mathrm{II}\right)}\right]$$

Moreover, roll torque for Type III can be expressed as follows:

(38) $$T_{1(\mathrm{III})}=R\mu_{1}\left[P_{\left(\mathrm{III}\right)}-\frac{2Klh\left(\delta^{+}\varepsilon_{f}+1\right)\left(\xi_{F}^{\delta^{+}+1}-1\right)}{\delta^{+}\Delta h\left(\delta^{+}+1\right)}+\frac{2KlQ_{F}}{\delta^{+}}\right]$$

(39) $$T_{2(\mathrm{III})}=-R\mu_{2}P_{\left(\mathrm{III}\right)}$$

The total roll torque per unit width is the sum of upper roll torque and lower roll torque.

### 2.7. Roll Power

Roll power of one roll can be calculated from peripheral velocity and torque of the roll. Owing to the difference in velocity and torque, the upper roll power per unit width $A_{1}$ and the lower roll power per unit width $A_{2}$ are always different in asymmetrical rolling.

(40) $$A_{1}=T_{1}\frac{v_{1}}{R}$$

(41) $$A_{2}=T_{2}\frac{v_{2}}{R}$$

In the plastic deformation of the strip, tension also plays an important role. The power of front tension or back tension, which is equal to the powers of the coiler or the uncoiler, is the product of tension and corresponding strip velocity. So, the powers per unit width of front tension and back tension can be expressed as follows:

(42) $$A_{f}=\sigma_{f}hv_{h}$$

(43) $$A_{b}=-\sigma_{b}Hv_{H}$$

The required power per unit width in one pass is the sum of roll powers and tension powers.

## 3. Experiments and Simulations

**Figure 4:**
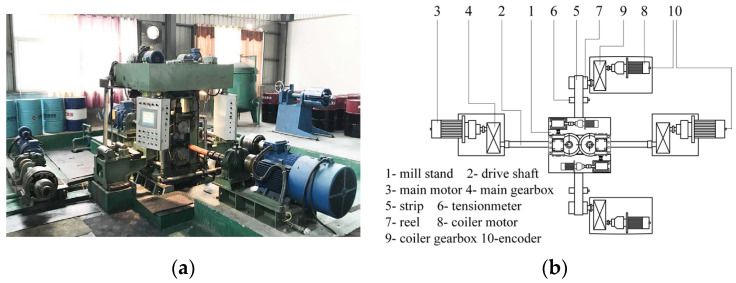
Asymmetrical mill for experimental rolling: (**a**) photo of the mill, (**b**) schematic of the top view of the rolling system.

Asymmetrical rolling experiments have been conducted on the four-high reverse mill as shown in Figure 4. This mill is equipped with the gauge meter, force transducer, tensiometer, encoder and lubrication system. Work rolls of the mill are separately driven. The nominal diameter and the barrel lengths of work roll are 90 mm and 220 mm, respectively. A maximum roll force of 800 kN can be afforded by the screw-down system.

In experiments, the work roll diameters are 88.0 mm, roll speed of the slow roll (lower roll) is 4.0 m/min, and the roll speed of the fast roll is adjusted on the basis of speed ratio and roll speed of the slow roll. Annealed 430 stainless steel strips of 0.5 mm in thickness and 80 mm in width were rolled with emulsion lubrication.

Roll force and roll torque were measured in experiments, 30 points were collected for each parameter and the average was used as experimental value.

The proposed models are used to simulate the multi-pass asymmetrical rolling of the strip, and roll pressure, three zone percentages, roll force, roll torque, roll power, rolling time and total work are calculated in the simulation. The roll diameter and strip width used in the simulation are the same as in the experiment. The roll speed of the slow roll (the lower roll) is set to 1.0 m/s ($v_{2}$ = 1.0 m/s), and the roll speed of the fast roll is determined via speed ratio ($v_{1}=iv_{2}$). The deformation resistance curve of the 430 stainless steel strip was obtained from tensile test results:

(44) $$K=\frac{2}{\sqrt{3}}\left(128.63+579.23{\bar{\varepsilon }}^{0.247}\right)$$

where $\bar{\varepsilon }=1-\left(0.4H+0.6h\right)/H_{0}$ is the average strain and $H_{0}$ is the initial thickness of the strip.

Four sets of simulations were carried out. In the first to the third set, strips of 1.0 mm thickness were successively rolled for 5 passes with the same reduction ratio of 30% per pass, and a different speed ratio was used for each strip in the same set. The difference between sets are front tension and back tension used. In the fourth set, strips of 0.6 mm thickness were successively rolled to a final thickness of 0.1 mm with the same roll force (roll force per unit width of 1.5 kN/mm) and different speed ratios.

## 4. Results and Discussions

In Discussions, speed ratio of 1.0 is used to represent the symmetrical rolling.

### 4.1. Analysis of Three Zone Percentages

**Figure 5:**
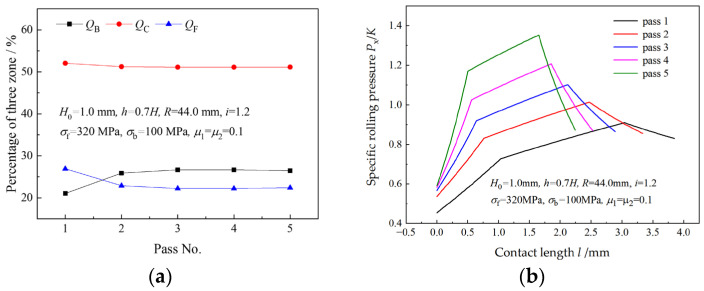
Variations of (**a**) three zone percentages and (**b**) specific roll pressure in five passes.

Three zone percentages and specific roll pressures in different passes when the speed ratio is 1.2 are illustrated in Figure 5. As can be seen from Figure 5a, the rolling pass has little effect on the three zone percentages when the speed ratio is unchanged. The deformation resistance of the strip leaps in the second pass due to strain-hardening, resulting in evident changes of $Q_{B}$ and $Q_{F}$ from the first pass to the second pass. As the pass increases, the contact length decreases obviously due to the decrease in the thickness reduction, resulting in a shorter platform in the distribution curve of the roll pressure and a higher average roll pressure. The platform in the roll pressure curve is caused by the cross-shear zone in the deformation region.

**Figure 6:**
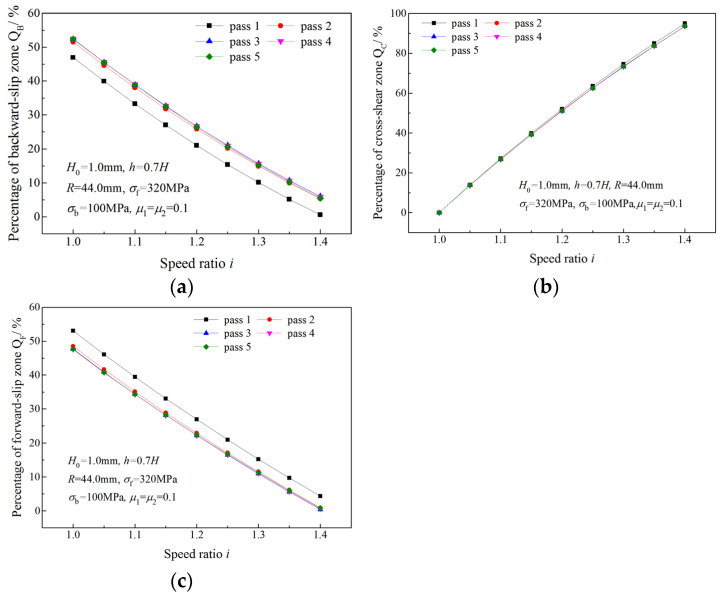
Effects of speed ratio on (**a**) percentage of backward-slip zone, (**b**) percentage of cross-shear zone and (**c**) percentage of forward-slip zone in five passes.

Figure 6 shows the effects of the speed ratio on three zone percentages in five passes. In the same pass, $Q_{B}$ and $Q_{F}$ decrease with an increasing speed ratio, and $Q_{C}$ increases with speed ratio. For the same speed ratio, $Q_{C}$ is almost the same in all passes, but $Q_{F}$ and $Q_{B}$ are almost unchanged in the latter four passes. A greater $Q_{F}$ and a lesser $Q_{B}$ in the first pass result from the much lower deformation resistance of the strip in the first pass.

**Figure 7:**
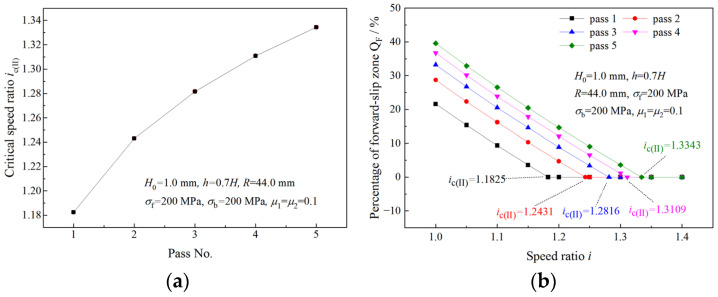
(**a**) Variation of the critical speed ratio with pass and (**b**) effects of speed ratio on the percentage of forward-slip zone.

**Figure 8:**
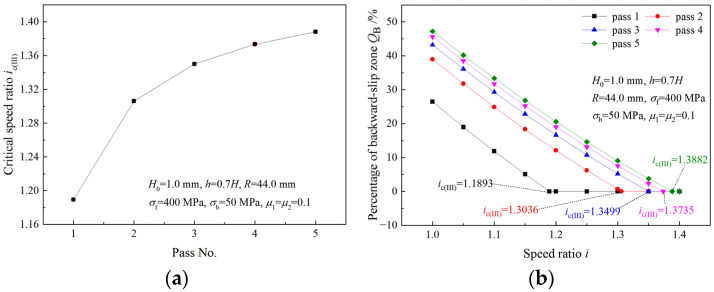
(**a**) Variation of critical speed ratio with pass and (**b**) effects of speed ratio on backward-slip zone percentage.

As shown in Figure 6a,c, *Q*_B_ and *Q*_F_ decrease with an increasing speed ratio in the same pass. When *Q*_F_ or *Q*_B_ becomes zero, the critical speed ratio is obtained. Figure 7 illustrates critical speed ratios in different rolling passes and the variation of *Q*_F_ with the speed ratio. When the speed ratio is less than the critical speed ratio, *Q*_F_ decreases with an increasing speed ratio; when speed ratio is greater than the critical speed ratio, *Q*_F_ become zero. The critical speed ratio is higher in latter passes. When the speed ratio reaches the critical speed ratio *i*_c(II)_, the deformation region type changes from Type I to Type II due to the disappearance of the forward-slip zone. A similar trend is found for *Q*_B_ and *i*_c(III)_ in Figure 8 when the deformation region type changes from Type I to Type III.

### 4.2. Analysis of Roll Force

**Figure 9:**
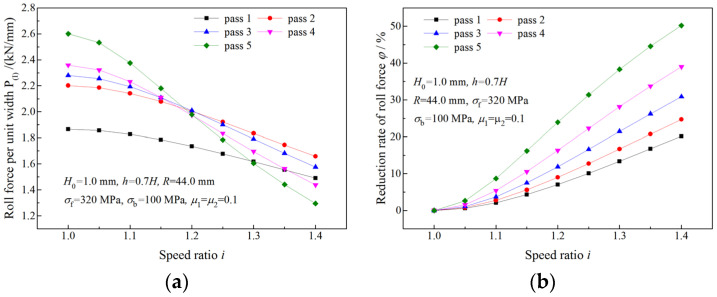
Effects of speed ratio on (**a**) roll force and (**b**) reduction rate of roll force when the deformation region type is Type I.

Figure 9 illustrates the effect of the speed ratio on the roll force when the deformation region is Type I. As shown in Figure 9a, less roll force is required by a rolling with a higher speed ratio in the same pass, especially in the fifth pass. A reduction rate of roll force, $\varphi_{i}$, is defined to evaluate the effect of the speed ratio *i* on roll force reduction:

(45) $$\varphi_{i}=\frac{P_{10}-P_{i}}{P_{10}}\times 100\%$$

where $P_{i}$ is the roll force per unit width required by the asymmetrical rolling with a speed ratio *i*, $P_{10}$ is the roll force per unit width required by symmetrical rolling. A greater $\varphi_{i}$ means the roll force can be more effectively reduced. It can be seen from Figure 9b that the maximum reduction rate, which is caused by asymmetrical rolling with the highest speed ratio, is found in the fifth pass. Curves in Figure 9 indicate that the asymmetrical rolling is especially suitable for the production of thin and hard strips.

**Figure 10:**
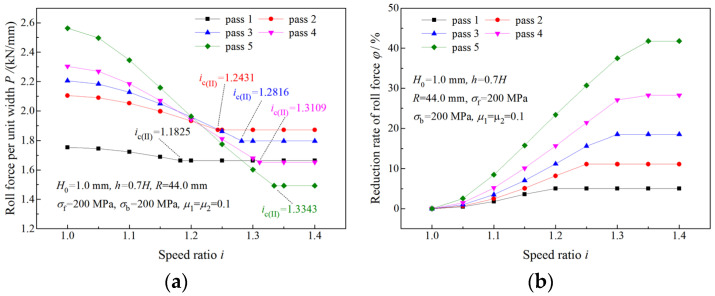
Effects of speed ratio on (**a**) roll force and (**b**) reduction rate of roll force when the deformation region type changes from Type I to Type II.

**Figure 11:**
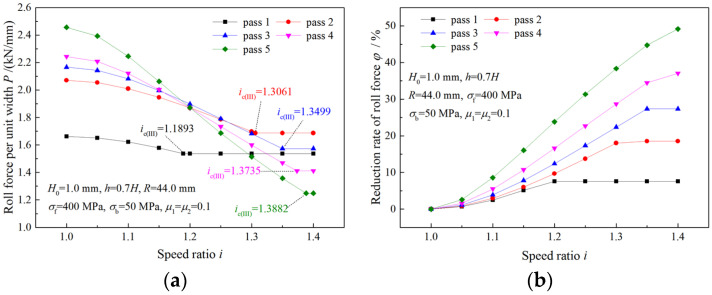
Effects of speed ratio on (**a**) roll force and (**b**) reduction rate of roll force when the deformation region type changes from Type I to Type III.

The variation of the roll force at the time of a deformation region type change is illustrated in Figure 10 and Figure 11. When the speed ratio is less than the critical speed ratio, a higher speed ratio results in less roll force (Figure 10a and Figure 11a) and a greater reduction rate of roll force (Figure 10b and Figure 11b). When the speed ratio is greater than the critical speed ratio, the roll force and reduction rate of roll force keep constant.

### 4.3. Analysis of Roll Torque

**Figure 12:**
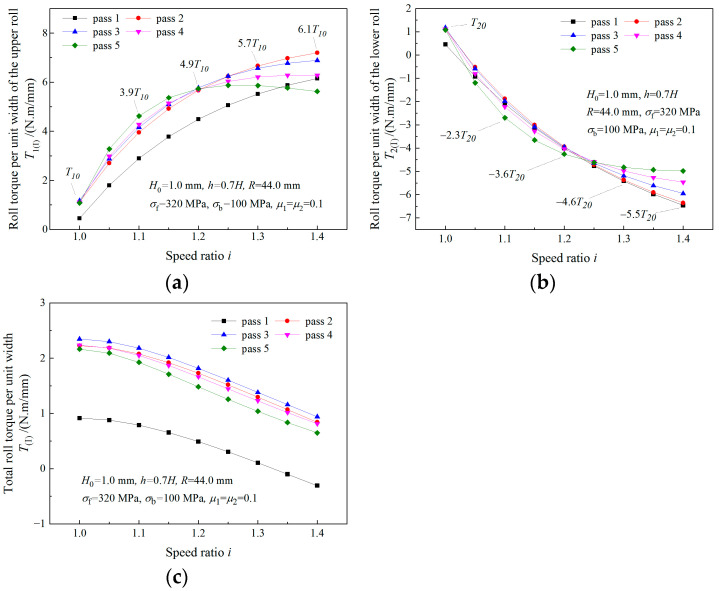
Effects of speed ratio on (**a**) upper roll torque, (**b**) lower roll torque and (**c**) total roll torque when deformation region is Type I.

As can be seen from Figure 12a,b, absolute values of both the upper roll and the lower roll increase with the increase in the speed ratio. Less total roll torque is required when a higher speed ratio is adopted (Figure 12c). The maximum absolute values of the required roll torque of the upper roll and the lower roll in symmetrical rolling are denoted by $T_{10}$ and $T_{20}$. Multiples of $T_{10}$ and $T_{20}$ are used to represent maximum absolute torques in asymmetrical rolling. Maximum absolute values of $6.1T_{10}$ and $5.5T_{20}$ are found for the upper roll and the lower roll in asymmetrical rolling with a speed ratio of 1.4. The roll torque of the lower roll in asymmetrical rolling is negative, which indicates that the lower drive motor is working as a generator.

**Figure 13:**
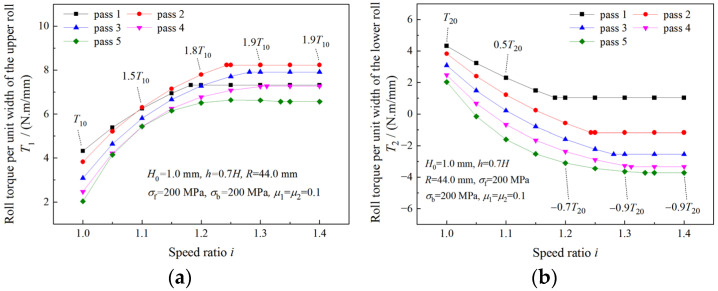
Variations of (**a**) upper roll torque and (**b**) lower roll torque with speed ratio when the deformation region changes from Type I to Type II.

**Figure 14:**
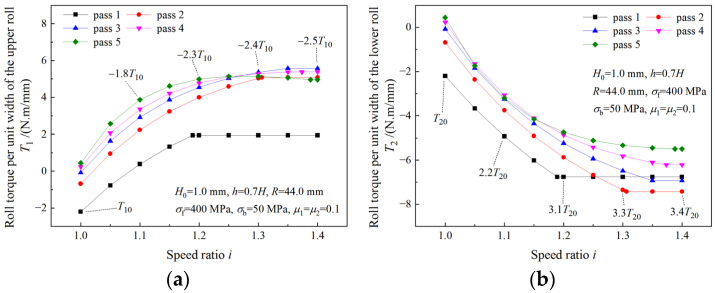
Variations of (**a**) upper roll torque and (**b**) lower roll torque with speed ratio when the deformation region changes from Type I to Type III.

The variations of roll torque with speed ratio in the case of deformation region type change are illustrated in Figure 13 and Figure 14. As the speed ratio increases within the limit of the critical speed ratio, the upper roll torque increases, while the lower roll torque decreases. When speed ratio is beyond the critical speed ratio, both the upper roll torque and the lower roll torque remain unchanged.

The negative upper roll torque in the first pass (Figure 14a) results from the roller die drawing effect caused by high front tension.

### 4.4. Analysis of Roll Power

**Figure 15:**
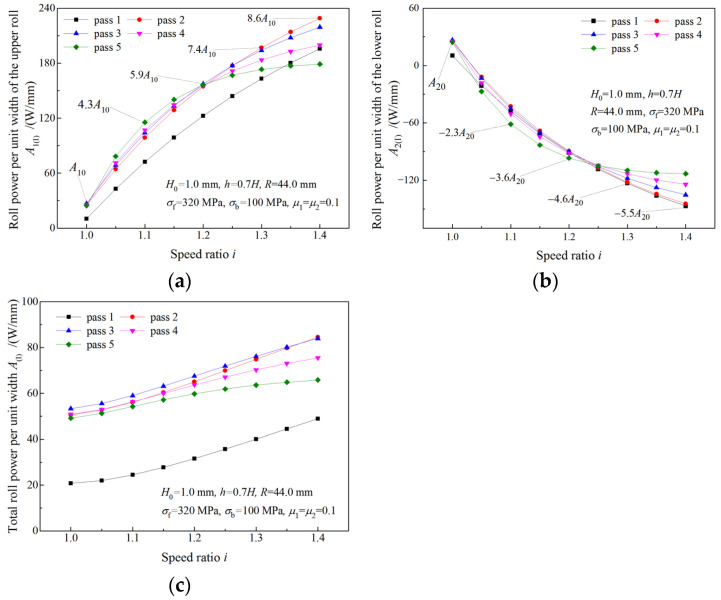
Effects of speed ratio on (**a**) roll power of upper roll, (**b**) roll power of lower roll and (**c**) total roll power when deformation region type is Type I.

The variations of roll powers with the speed ratio are illustrated in Figure 15. As the speed ratio increases, roll power of the upper roll (the fast roll) increases (Figure 15a), while roll power of the lower roll (the slow roll) decreases (Figure 15b). The total roll power increases more slowly with the speed ratio than the roll power of the upper roll (Figure 15c). The maximum required roll powers of the upper roll and the lower roll in symmetrical rolling are denoted by $A_{10}$ and $A_{20}$, and the maximum absolute values of the required roll powers in asymmetrical rolling are represented by multiples of $A_{10}$ and $A_{20}$. The required roll power of the upper roll is much higher in asymmetrical rolling than that in symmetrical rolling. When the strip is rolled with speed ratio 1.4, a maximum roll power of $8.6A_{10}$ is obtained for the upper roll (Figure 15a). Thus, more powerful drive motors are required in asymmetrical rolling. The negative roll power of the lower roll in asymmetrical rolling indicates that the lower drive motor is working in a generation state.

**Figure 16:**
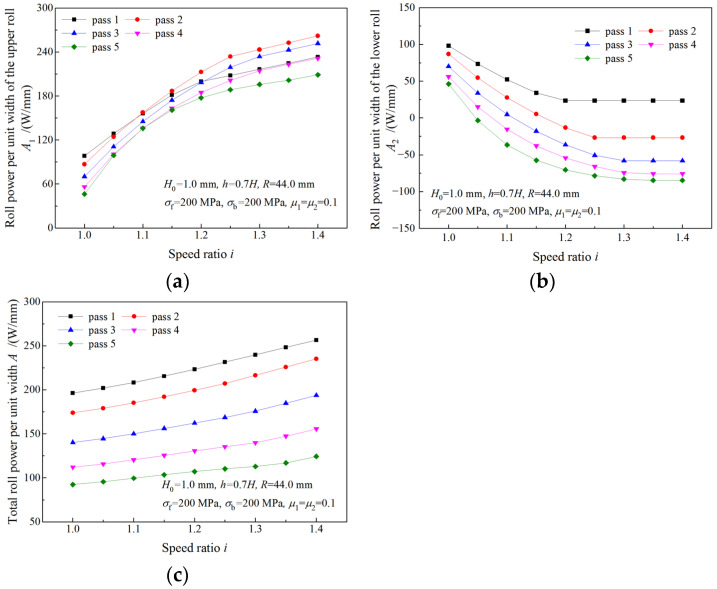
Effects of speed ratio on (**a**) roll power of upper roll and (**b**) roll power of lower roll and (**c**) total roll power when deformation region changes from Type I to Type II.

The variation of roll power when the deformation region changes from Type I to Type II is illustrated in Figure 16. As can be seen from Figure 16a,c, both upper roll power and total roll power increase with the speed ratio. When the speed ratio increases within the limit of the critical speed ratio, the roll power of the lower roll decreases. When the speed ratio is beyond the critical speed ratio, the roll power of the lower roll remains unchanged (Figure 16c). Due to the increase in the roll speed, the upper roll power continues increasing after the speed ratio reaches the critical speed ratio.

### 4.5. Analysis of Total Work and Rolling Time

In order to investigate the effect of speed ratio on time consumption and energy consumption in strip manufacturing, a simulation for multi-pass rolling with the same roll force (roll force per unit width of 1.5 kN/mm) was carried out. Strips of 0.6 mm in thickness and 80 mm in width are rolled to the thickness of 0.1 mm in the simulation. Each strip is rolled with a different speed ratio.

**Figure 17:**
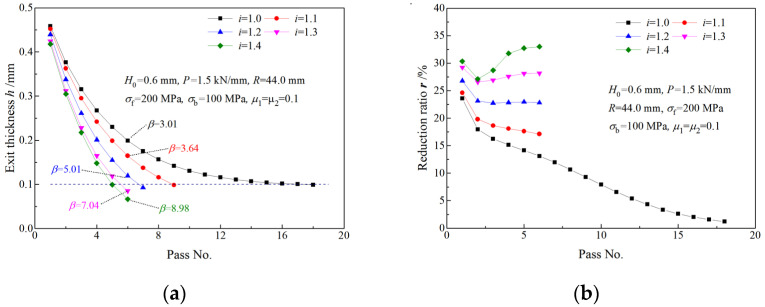
Variations of (**a**) exit thickness and (**b**) thickness reduction ratio with rolling pass under different speed ratio.

As can be seen from Figure 17a and Table 1, fewer rolling passes are required by rolling with a higher speed ratio to attain the same exit thickness. Only 6 passes are required when rolling with a speed ratio of 1.4 to attain an exit thickness of 0.1 mm while 18 passes are required by symmetrical rolling. A greater reduction ratio can be obtained by rolling with a higher speed ratio and the same roll force, and reduction ratios obtained by symmetrical rolling in later passes gradually approach zero (Figure 17b). An elongation coefficient, *β*_i_ = *H*_0_/*h*, is employed to evaluate the effect of speed ratio *i* on thickness reduction. The elongation coefficient after the sixth pass for a speed ratio of 1.4 (8.98) is much greater than that for symmetrical rolling (3.01), which indicates that the thickness reduction efficiency can be improved evidently by increasing the speed ratio.

In the calculation of time and work, the initial length of the strip is $L_{0}$ = 1000 m, the initial thickness is $H_{0}$ = 0.6 mm, the final exit thickness is *h* = 0.1 mm, the strip width is 80 mm and the exit speed strip is *v*_h_ =100 m/min.

**Figure 18:**
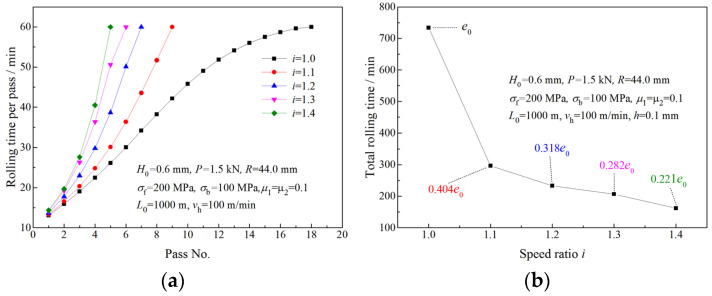
Effects of speed ratio on rolling time: (**a**) rolling time in a pass, (**b**) total rolling time for the same initial and final thickness.

The rolling time per pass increases with the rolling pass, and the increase is more obvious with a higher speed ratio (Figure 18a). The total rolling time required for the strip being rolled from 0.6 mm to 0.1 mm via symmetrical rolling is denoted by $e_{0}$. Owing to fewer roll passes being required to attain the final thickness by asymmetrical rolling (Figure 17a), the total rolling time required by asymmetrical rolling is much shorter than that required by symmetrical rolling (Figure 18b). This result suggests that asymmetrical rolling can provide great time savings in strip production.

**Figure 19:**
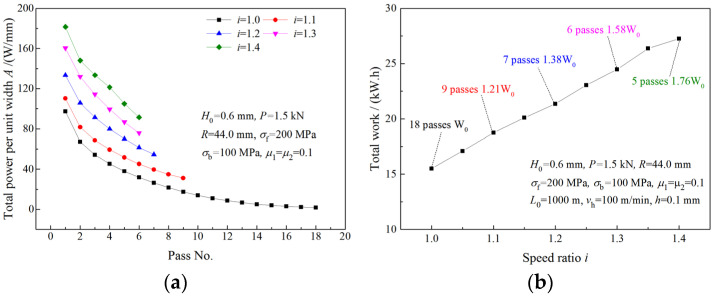
Effects of speed ratio on (**a**) the power per unit width and (**b**) total work.

Figure 19a illustrates the total power per unit width required for the strip being rolled from 0.6 mm to 0.1 mm with the same roll force per pass. The total work consumed in the rolling process is calculated from the total power per unit width and the rolling time per pass. As can be seen from Figure 19b, *W*_0_ is the total work consumed by symmetrical rolling; though fewer rolling passes are required, more work is consumed by asymmetrical rolling.

### 4.6. Verification of Models

Analytical values of roll force and roll torque were compared with measured experimental values to verify the validity of the proposed models.

**Figure 20:**
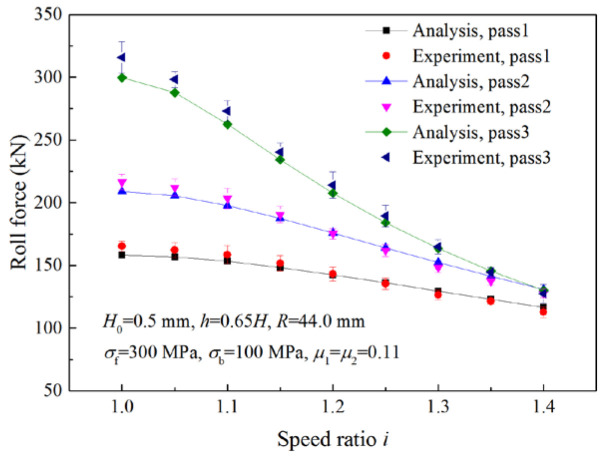
Comparisons of roll forces between analytical and experimental results in three passes.

Figure 20 shows the comparison between the calculated roll forces and the experimental results in three passes. Strips were successively rolled for three passes with a reduction ratio of 35% per pass and a different speed ratio for each strip. As can be seen from the figure, both analytical and experimental roll forces decrease with the increase in the speed ratio, and the reduction in the roll force is more evident in the third pass. The maximum error between the experimental results and analytical values is 5.40%.

**Figure 21:**
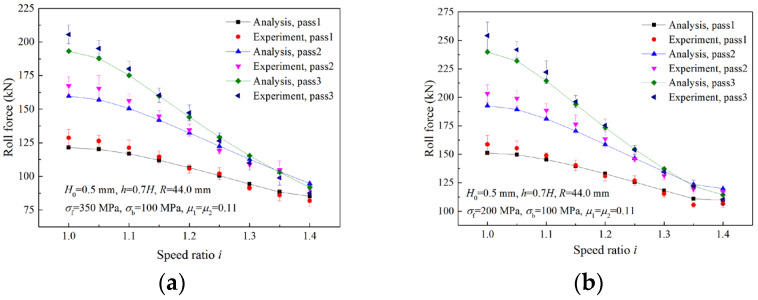
Comparisons between analytical roll forces and experimental roll forces with different front tensions: (**a**) $\sigma_{f}$ = 350 MPa, (**b**) $\sigma_{f}$ = 200 MPa.

Figure 21 illustrates the comparisons between analytical roll forces and experimental results for two different front tensions. When the front tension is 350 MPa, the deformation region type is Type I, and both the analytical roll force and experimental roll force decrease with the increase in the speed ratio. When the front tension is reduced to 200 MPa, the critical speed ratio is reached at the first pass. As the speed ratio increases within the critical speed ratio, both analytical and experimental roll forces decrease. Both analytical and experimental roll forces remain unchanged when the speed ratio is beyond the critical speed ratio. The maximum errors of roll force between experimental results and analytical values are 5.98% and 6.41%, respectively, for the two front tensions.

**Figure 22:**
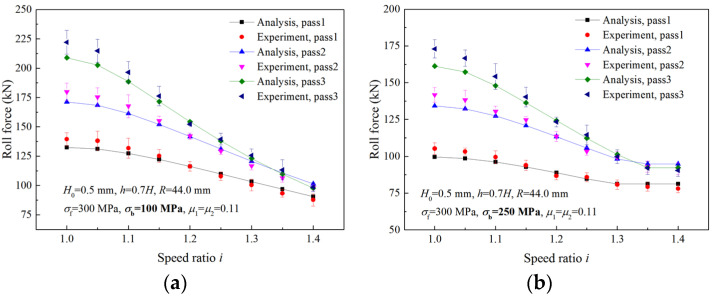
Comparisons of roll forces between analytical and experimental results with different back tensions: (**a**) $\sigma_{b}$= 100 MPa, (**b**) $\sigma_{b}$= 250 MPa.

Figure 22 shows the comparison between analytical values and experimental results of the roll force in three passes for two different back tensions. When $\sigma_{b}$ = 100 MPa, the deformation region type is Type I in all passes, and both analytical values and experimental results of the roll force decrease with the increase in the speed ratio. When the back tension is increased to 250 MPa, the forward-slip zone disappears under a high speed ratio, resulting in the change of the deformation region type from Type I to Type II. Both analytical roll forces and experimental roll forces first decrease with the increase in the speed ratio and then remain unchanged after the critical speed ratio is reached. The maximum errors between experimental results and analytical values of roll force are 6.34% and 7.24%, respectively, for the two back tensions.

**Figure 23:**
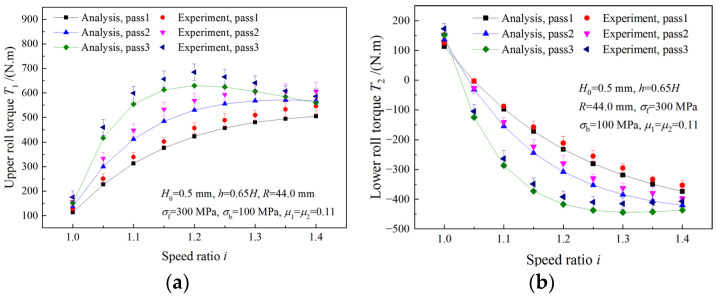
Comparison between analytical roll torque and experimental roll torque: (**a**) the upper roll torque, (**b**) the lower roll torque.

The comparison of analytical roll torques and experimental roll torques in three passes are shown in Figure 23. The same trends of experimental results and analytical values are found for both the upper roll torque and the lower roll torque. With the increase in the speed ratio, the upper roll torque increases, while the lower roll torque decreases. The maximum errors of upper roll torque and lower roll torque are 15.92% and 13.95%, respectively.

## 5. Conclusions

Mathematic models for asymmetrical rolling were developed by employing the slab method to simulate the multi-pass asymmetrical rolling process of strip. The roll pressure, roll force, roll torque and roll power were calculated in the simulation. Characteristics of mechanical parameters were analyzed on the basis of simulation results. Experimental rolling was conducted to verify the accuracy of the proposed models. The following conclusions are obtained:

- Good consistency between the analytical results and the experimental measurements suggests excellent accuracy of the proposed models.
- Less roll force is required by asymmetrical rolling to obtain the same thickness reduction than that by symmetrical rolling. The required roll force decreases with the increase in the speed ratio until the critical speed ratio is reached; then the required roll force remains constant. A greater reduction rate of roll force is found in the last pass of a rolling schedule, which indicates that asymmetrical rolling is especially suitable for thin and hard strips.
- The upper roll torque first increases with the increase in the speed ratio, and the lower roll torque first decreases with the increase in the speed ratio; then both of them become unchanged when the critical speed ratio is reached. The roll powers show a similar trend, but the upper roll power does not keep constant after the critical speed ratio is reached due to the increase in the upper roll speed. The lower roll torque and lower roll power are always negative in asymmetrical rolling, which means the lower driver motor is working as a generator.
- Less roll force is required by asymmetrical rolling than that by symmetrical rolling in the same pass, but much greater upper roll torque and upper roll power are required by asymmetrical rolling; thus, stronger drive shafts and more powerful drive motors are required in asymmetrical rolling.
- A greater reduction ratio can be obtained by asymmetrical rolling than that by symmetrical rolling with the same roll force. Thus, fewer passes and, consequently, less total rolling time are required to obtain the same final thickness by asymmetrical rolling. The required total rolling time decreases with the increase in the speed ratio, but more total work is required by the higher speed ratio. Rolling time can be saved with asymmetrical rolling at the cost of more energy consumption.

## Figures and Tables

**Table 1:** The final thickness and number of passes under different speed ratio.

Speed Ratio *i*	Number of Passes	Final Thickness/mm
1.0	18	0.099
1.1	9	0.099
1.2	7	0.093
1.3	6	0.085
1.4	6	0.067

## Data Availability

Not applicable.
